# Reliable cell retention of mammalian suspension cells in microfluidic cultivation chambers

**DOI:** 10.1038/s41598-023-30297-5

**Published:** 2023-03-08

**Authors:** Julian Schmitz, Birgit Stute, Sarah Täuber, Dietrich Kohlheyer, Eric von Lieres, Alexander Grünberger

**Affiliations:** 1 Multiscale Bioengineering, Faculty of Technology, Bielefeld University, Universitätsstraße 25, 33615 Bielefeld, Germany; 2 Center for Biotechnology (CeBiTec), Bielefeld University, Universitätsstraße 27, 33615 Bielefeld, Germany; 3 IBG-1: Biotechnology, Forschungszentrum Jülich, Wilhelm-Johnen-Straße 1, 52428 Jülich, Germany; 4 Microsystems in Bioprocess Engineering, Institute of Process Engineering in Life Sciences, Karlsruhe Institute of Technology (KIT), Fritz-Haber-Weg 2, 76131 Karlsruhe, Germany

**Keywords:** Biophysics, Biotechnology, Engineering

## Abstract

Microfluidic cultivation, with its high level of environmental control and spatio-temporal resolution of cellular behavior, is a well-established tool in today’s microfluidics. Yet, reliable retention of (randomly) motile cells inside designated cultivation compartments still represents a limitation, which prohibits systematic single-cell growth studies. To overcome this obstacle, current approaches rely on complex multilayer chips or on-chip valves, which makes their application for a broad community of users infeasible. Here, we present an easy-to-implement cell retention concept to withhold cells inside microfluidic cultivation chambers. By introducing a blocking structure into a cultivation chamber’s entrance and nearly closing it, cells can be manually pushed into the chamber during loading procedures but are unable to leave it autonomously in subsequent long-term cultivation. CFD simulations as well as trace substance experiments confirm sufficient nutrient supply within the chamber. Through preventing recurring cell loss, growth data obtained from Chinese hamster ovary cultivation on colony level perfectly match data determined from single-cell data, which eventually allows reliable high throughput studies of single-cell growth. Due to its transferability to other chamber-based approaches, we strongly believe that our concept is also applicable for a broad range of cellular taxis studies or analyses of directed migration in basic or biomedical research.

## Introduction

Microfluidic cultivation (MC) of cells under highly controllable environmental conditions is a well-established operation in today’s microfluidics^1^. Here, microfluidic single-cell cultivation (MSCC) with its focus on analyzing single-cell behavior represents a specific subcategory to the field^2^. Typically, MSCC of various single-celled organisms can be realized by the application of cultivation chambers, where cells are trapped in designated regions and cultivated as monolayer colonies for distinct analytical investigations^3^. Due to the spatial restrictions of the cultivation chambers, cells are retained inside the desired compartment and can be analyzed by live cell imaging, resulting in a high temporal resolution of single-cell behavior. With this setting, a broad range of cell types, ranging from algae over bacteria to fungi and mammalians, can be cultivated^4–10^.

Commonly, cells inside these cultivation chambers are supplied with nutrients by diffusive mass exchange. For this purpose, the cultivation chambers are arranged along supply channels in which laminar flow of cultivation medium prevails^3^. At the same time supply channels do not only serve for nutrient supply but also represent a potential way for cells to escape the cultivation chambers. Therefore, design and dimension of supply channel, cultivation chamber, and the chamber’s entrance always represents a tradeoff between optimal nutrient supply and sufficient cell retention. Especially for the long-term cultivation of slow growing cells^11^ as well as the microfluidic cultivation of motile cells^12^, a reliable cell retention concept is a fundamental requirement to prevent permanent cell loss, which otherwise compromises qualitative and quantitative cell studies.

In the last years, MSCC for mammalian cell lines, in particular for Chinese hamster ovary (CHO) cells, became of increasing interest to investigate growth and robustness of industrially relevant production cell lines^13,14^. Here, especially suspension cells are of commercial relevance, as large-scale bioproduction processes exclusively use suspension cell lines^15,16^. In comparison to bacteria and yeast, which are traditionally kept inside the cultivation chambers by squeezing them tightly into narrow chamber heights, CHO cells cannot be retained inside the chamber the same way as cells with rigid cell walls because of their deformable nature. Although they are not able to move actively, when cultivated in MSCC devices CHO suspension cells also randomly migrate inside the boundaries of the cultivation chambers^13,17^. Unfortunately, these random movements frequently lead to cell loss, as randomly migrating cells leave the chamber through the entrance. Additionally, squeezing cells inside narrow microfluidic structures potentially influences cellular behavior because of spatial restriction and thereby could lead to compromised growth. Thus, other approaches to reliably retain cells must be established to permit quantitative analysis of single-cell cultivation.

In order to overcome this challenge, a variety of microfluidic devices with different approaches to retain (motile) cells have been published over the years (Table 1). Several of these setups rely on PDMS multilayer chips to either withhold cells at the bottom of a cultivation well^18,19^ or to establish on-chip valving to lock cultivation chambers after cells have been trapped^20–22^. Others exhibit cultivation chambers made of SU-8, that are combined with fluid control layers made of PDMS-membrane hybrids to ensure sufficient supply of the cultivated cells with medium^23^. Although sophisticated and efficient, all these approaches are very complex and prone to malfunction, since their setup depends on a multitude of fabrication steps and technical components.

**Table 1:** Overview of recently published microfluidic cultivation devices with enhanced cell retention. The listed approaches are itemized concerning the characteristics of the respective cultivation area, the cell retention method, and the nutrient supply. Additionally, the cultivated cell type is indicated.

Cultivation area	Retention method	Nutrient supply	Cultivated cell type	References
Wells	Sedimentation	Diffusive	Mouse hematopoietic cells	Lecault et al.^18^
Wells	Sedimentation	Diffusive	Mouse ovarian cancer cells	Dadgar et al.^19^
Chambers	Pneumatic valves	Convective	*Chlamydomonas reinhardtii*	Eu et al.^20^
Chambers	Pneumatic valves	Diffusive	Primary murine cells, Murine embryonic stem cells	Dettinger et al.^21^
Chambers	Pneumatic valves	Convective	Human primary mesenchymal stem cells	Gómez-Sjöberg et al.^22^
Chambers	Membrane sealing	Diffusive	*Dictyostelium discoideum*	Delince et al.^23^
Chambers	Air sealing	None	*Salpingoeca rosetta*	Halperin et al.^24^
Chambers	Narrow entrances	Diffusive	Chinese hamster ovary cells	Kolnik et al.^25^

In this respect, several microfluidic designs consisting of a PDMS single-layer chip have been introduced as well. Some rely on dead-end cultivation chambers which are sealed with air after cell loading, to retain cells inside the chamber^24^. While cell retention is highly reliable, environmental conditions in sealed cultivation chambers resemble a batch-mode cultivation and thus are subjected to drastic environmental changes over long-term cultivation and thereby disqualify these designs for cultivations where defined environmental control is desired. Other concepts rely on cultivation chambers with narrow entrances, minimizing the cross section between cultivation chamber and supply channels and thereby reducing the probability of cell loss^25^.

In comparison to previously published complex multilayer structures or non-sufficient single-layer designs, in this work we achieved cell retention by introducing a thin PDMS barrier into the cultivation chamber’s entrance. Thus, cell loss during long-term single-cell cultivation is effectively prevented while sufficient diffusive medium supply of the captured cells is still ensured.

## Results and discussion

### Design and fabrication

**Figure 1:**
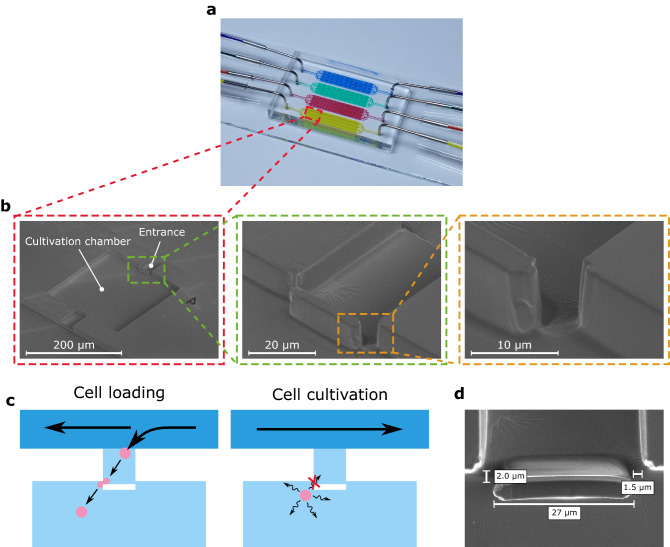
Structure and cell retention concept of the MSCC device with enhanced cell retention for CHO suspension cell lines. (**a**) Microfluidic PDMS-glass-based cultivation device. (**b**) Scanning electron microscopy image of the microfluidic structure illustrating the devices dimensions and trapping barrier. (**c**) Schematic drawing of the loading procedure and cell retention concept based on a PDMS barrier that is traversable by applying pressure during cell loading but non-traversable by random cellular movement during cultivation. (**d**) Scanning electron microscopy image of the PDMS barrier located in the cultivation chamber’s entrance.

In this work, we developed an easy-to-integrate PDMS barrier for our previously developed microfluidic cultivation device (Fig. 1a)^13^ that enables enhanced cell retention by introducing a physical blocking structure into the cultivation chamber’s entrances (Fig. 1b), that only can be passed by applying pressure during cell loading but does not permit the escape of randomly moving CHO cells during cultivation (Fig. 1c). Here, pressure-induced deformation of cells is assumed to be the decisive factor in cell loading, yet slight bending of the PDMS barrier might additionally promote this process. As can be seen in Fig. 1b, the barrier structure exhibits the same height as the chamber, so that it fully locks up the entrance and does not function as a movable hatch. With a width of 27 µm the barrier nearly closes the whole entrance except for a 1.5 µm wide and 2.0 µm high gap at both sides (Fig. 1d).

The compatibility of the PDMS barrier with our previously developed MSCC design ensures simple chip fabrication and easy application for the cultivation of CHO suspension cells. The microfluidic cultivation device holds four identical cultivation arrays, each consisting of four parallel supply channels. Every cultivation array exhibits in total 60 cultivation chambers, 30 of them are arranged in line between two supply channels. To ensure monolayer growth of trapped cells, the modified design holds a limited chamber height of approx. 10 µm (Supplementary Fig. S1A). Based on our previous design, the ratio between supply channel and cultivation chamber height remained constant (approx. 2:1) to restrict laminar flow to the supply channels, which results in exclusively diffusive mass exchange between channel and chamber. Likewise, the supply channel width of 200 µm and the cultivation chamber’s base area of 200 × 200 µm^2^ stayed unchanged since it allows MSCC experiments for up to 7 days until cells outgrow the limited space (Supplementary Fig. S1B,C).

Nearly closing the whole entrance of the cultivation chamber makes seeding cells more difficult. Therefore, we applied air to the microfluidic cultivation device to create a directed flow through the cultivation chambers (Supplementary Fig. S2). Air bubbles can be introduced to the cultivation array through the inlet by almost completely withdrawing the cell suspension again, so that the supply channels are again filled with air like right before priming the device with cell suspension. However, this procedure must be conducted very carefully, otherwise cells will be sheared when passing through the narrowing between barrier and cultivation chamber wall (Supplementary Video S1). Additionally, no air bubble must remain inside the microfluidic cultivation device during the subsequent perfusion, or the intended flow profile will be disturbed. Performing these steps with approx. 1000 cells led to a representative viability of 89.3% subsequent to cell loading (Supplementary Fig. S3).

Although introducing air helps with maneuvering cells into the cultivation chambers, yet it is not possible to guarantee single-cell loading for every individual chamber of a cultivation array. In an exemplary loading experiment, four individual cultivation arrays were inoculated with a loading cell density of 3.59 × 10^6^ cells mL^−1^. Out of the 240 potentially loadable cultivation chambers 62.5% showed one or more cells after executing the above-described loading procedure whereas 37.5% stayed empty (Supplementary Fig. S4). As loading of the device is performed manually and the presented design does not feature any special structures to guide cells into the cultivation chambers, seeding cells is a random process. Therefore, efficiency of the loading procedure depends on the cell density of the inoculation culture as well as the expertise of the operator. Here, 25% of all loaded chambers showed only one trapped cell, while the frequency of occurrence of chambers with two to five cells per chamber decreased gradually (Supplementary Fig. S5). In general, when cell density is high, cells are trapped in nearly every of the available chambers, however most likely only few chambers will contain a single cell. When cell density is low, only a few chambers will contain cells at the end of the loading procedure, though the likelihood of seeding just one cell into a chamber increases. Repeating the loading procedure multiple times additionally effects the number of loaded cells per chamber as well as the number of populated chambers per cultivation array. Consequently, adapting one's loading procedure to the experiments' objective is essential. In case of MSCC in the context of heterogeneity studies only cultivation chambers with one initial cell are defined as relevant, as this guarantees an isogenic population with one progenitor cell. For studying e.g., cellular migration behavior or cell-to-cell interaction on the single-cell level cultivation chambers with more than one initial cell are feasible as well.

### Microfluidic characterization

**Figure 2:**
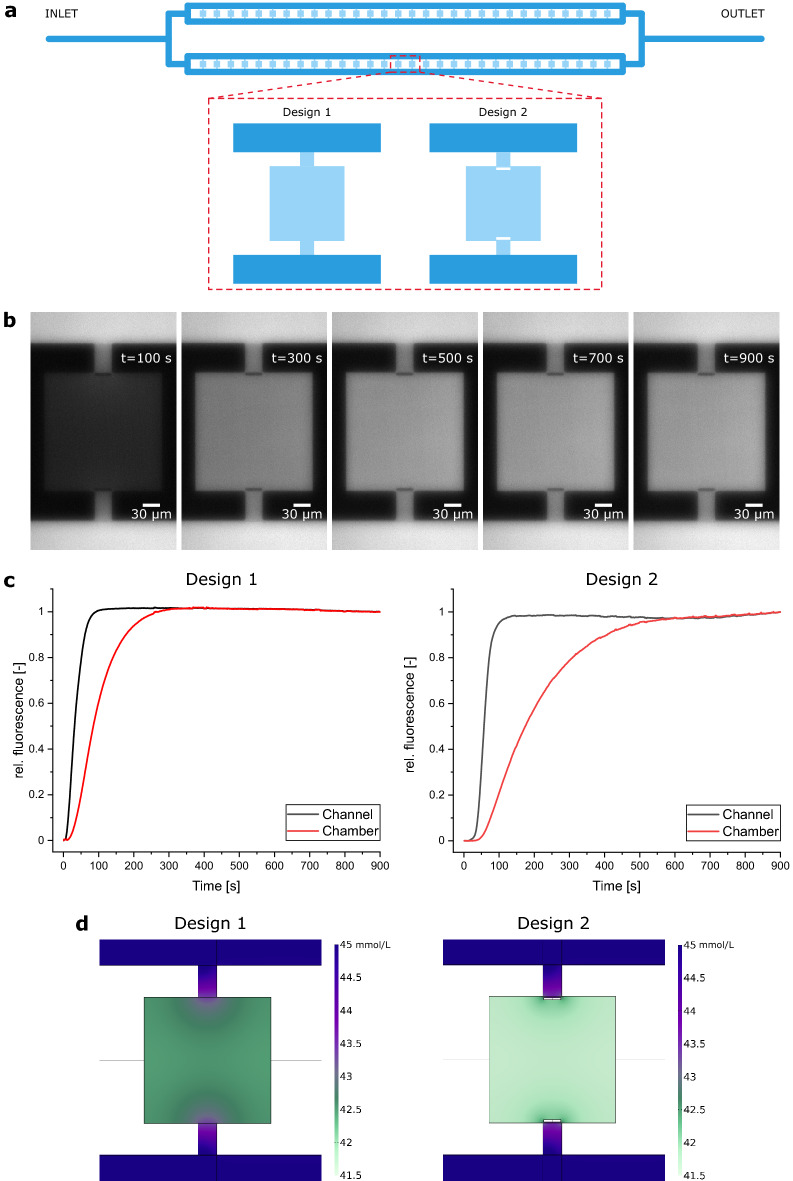
Microfluidic characterization of the MSCC designs. (**a**) Individual cultivation array that contains 60 cultivation chambers with either our previous chamber design (Design 1)^13^ or the chamber design from this work (Design 2). (**b**) Image sequence of trace substance experiments to quantify diffusive mass exchange for the MSCC device with Design 2. (**c**) Medium exchange duration until full equilibrium between channel and chamber is achieved based on rel. fluorescein signal for both designs. (**d**) Glucose concentration profile during MSCC cultivation assuming a steady state with 181 cells inside the chamber with a constant glucose uptake rate of 3800 nmol per 10^6^ cells and day for both designs.

As a result of almost closing the cultivation chamber’s entrance, not only loading characteristics of our new microfluidic design are drastically changed, but also mass exchange between channel and chamber is decreased. During MSCC experiments environmental conditions relating to nutrient concentrations are kept constant due to steady perfusion of the cultivation device. However, as cells start to fill the chamber, limitations might occur when cellular uptake rate outruns diffusive mass exchange. Therefore, we quantified diffusive mass exchange as well as glucose concentration profiles inside the presented device by fluorescein trace substance experiments and computational fluid dynamics (CFD) simulations according to the model published by Schmitz et al.^13^ for our already established design (Design 1) and our newly developed design (Design 2) with enhanced cell retention capability (Fig. 2a).

Figure 2b and the respective video (Supplementary Video S2) show that the fluorescein signal inside the cultivation chamber of Design 2 increases clearly delayed to the fluorescence inside of the supply channels. As can be seen from the rel. fluorescence level on Fig. 2c it takes 600 s until the same medium conditions from the supply channels are present inside the cultivation chamber. This diffusive exchange duration is in accordance with additionally performed CFD simulations which illustrate a potential medium switch from 0 to 45 mM glucose (Supplementary Fig. S6). In comparison to our previously published MSCC device (Design 1), where equilibrium was reached after approx. 300 s (Supplementary Video S3, Fig. 2c), this is a delay of 100%. However, since our device is operated in a static way with constant cultivation conditions, no problems caused by the prolonged medium exchange duration are to be expected, since chambers are inoculated with only few cells and cellular growth with a division time of approx. 15 h is slow in comparison to the determined medium exchange duration (t_exchange_ = 600 s). Additionally, we analyzed the cultivation conditions concerning glucose concentration inside the cultivation chamber in this steady state by CFD simulations to prove that no limitations occur even in a completely overgrown chamber. Here, the results indicate that with approx. 180 cells inside a single cultivation chamber, which is a representative cell number per chamber after 150 h of cultivation, no glucose limitation arises, given that the minimal glucose concentration does not drop below 41.5 mmol L^−1^ (Fig. 2d). As CFD simulations were only performed for glucose, the decrease of other media components due to cellular metabolism and slower diffusion into the cultivation chamber might be more drastic. However, cultivation was performed in a constantly perfused operating mode using commercially high cell density medium. Therefore, any occurring limitation is highly unlikely and sufficient supply of retained cells is given for the whole cultivation time. If complete exchange of medium and thereby changing the cultivation conditions is desirable for other research questions than long-term cultivation, the clearly prolonged equilibration time has to be considered.

### Cell retention capability

**Figure 3:**
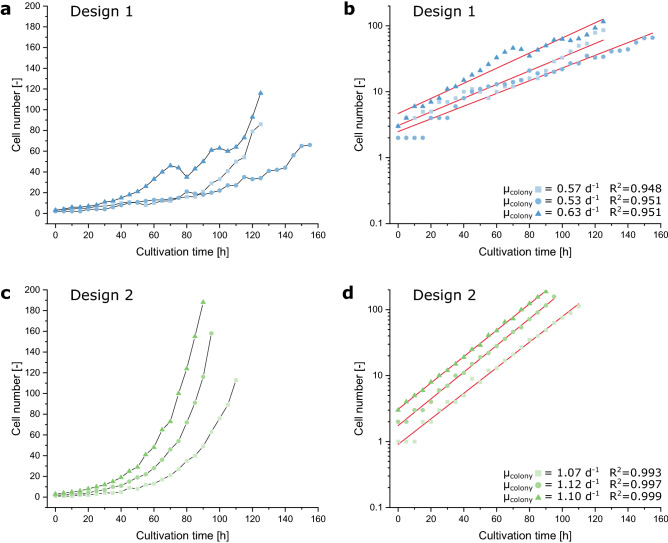
MSCC of CHO cells and cell retention assessment of the MSCC designs. (**a**) Growth of three CHO-K1 microcolonies from three individual cultivation arrays cultivated applying Design 1. (**b**) Semi-logarithmically plotted growth profile of the microcolonies cultivated applying Design 1. (**c**) Growth of three CHO-K1 microcolonies from two individual cultivation arrays cultivated applying Design 2. (**d**) Semi-logarithmically plotted growth profile of the microcolonies cultivated applying Design 2.

Multiple cultivation experiments with both cultivation chamber designs were performed to evaluate the cell withholding capability of the novel retention concept against the basic design. By quantifying the cell number during cultivation, we recorded the growth of three microcolonies for both designs. Figure 3a shows the growth progression of three microcolonies that were cultivated using Design 1. The growth curves of the three depicted microcolonies feature distinct bends in their progression, which clearly correlate with the loss of cells from the cultivation chambers. Looking at the respective semi-logarithmic plot illustrates the influence of dissipating cells on the specific growth rate, which is clearly decreased over the whole cultivation duration (Fig. 3b). In contrast, applying Design 2 results in more uniformly progressing growth curves (Fig. 3c). As the starting cell number varies between one and three cells, the rise of the curves is slightly delayed in time but for each microcolony strictly exponential in its character. Analyzing the specific growth rate resulting from the semi-logarithmic plot (Fig. 3d) shows highly reproducible values, which are twice as high as those obtained from Design 1.

This difference in cell retention, which is reflected in the curve progression of Fig. 3, and its influence on microcolony growth becomes apparent when looking at the corresponding videos (Supplementary Videos S4 and S5). With Design 2 cells only leave the chamber when they are pushed out by other cells or directly divide throughout the narrow gap between barrier and chamber wall, while cells in Design 1 frequently leave the cultivation chamber through the unlocked entrances because of their random movements. A negative effect on physiology or growth behavior caused by cell deformation during the loading procedure was not observed. As CHO cells are highly flexible, they return to their normal circular morphology quickly after the cell loading is completed. Thus, the loading procedure seems not to affect the cells permanently (Fig. 3, Supplementary Video S5).

**Figure 4:**
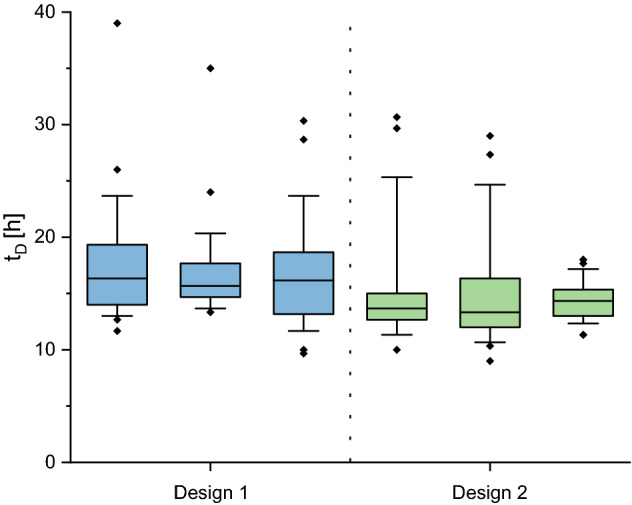
Comparison of single-cell division behavior between the two microfluidic cell retention concepts. Depicted are the single-cell doubling times t_D_ of cells cultivated in chambers with Design 1 (n = 29, 25, 24) and Design 2 (n = 25, 27, 30). The colored segment marks the interquartile range from 25 to 75%, the horizontal lines show the median. The whiskers represent the 10% and 90% percentile and the tilted squares mark rare cellular events outside the predefined percentiles.

Certainly, cell loss during long-term cultivation leads to a distinct underestimation of specific growth rates µ when determined on colony level and thereby results in immense differences between growth rate estimation on single-cell level (µ_single-cell_) and on colony level (µ_colony_)^13^. In order to have a closer look on this discrepancy, we additionally analyzed growth on the single-cell level by determining the doubling time t_D_ of cellular division events during the already presented cultivations. Here, the comparison between cells cultivated with both designs shows no significant difference (Fig. 4).

By assuming exponential growth, t_D_ can be converted into µ applying Eq. (1):

1 $$t_{D} = \frac{\ln (2)}{\upmu }$$

Comparing the above determined µ_colony_ with the average µ_single-cell_ of the same microcolonies, it is clearly noticeable that these two values show only small variations when cells are cultivated in the cultivation chamber with Design 2 (Table 2). However, cultivating cells under recurring cell loss with Design 1 leads to a noticeable difference.

**Table 2:** Comparison of the single-cell growth rate data µ_single-cell_, calculated from the geometrical mean of the determined single-cell doubling times t_D_, with the colony growth rate data µ_colony_, determined graphically based on the cell number.

Design	Replicate	$\overline{t}_{D}$, _geom_ (h)	µ_single-cell_ (h^−1^)	µ_colony_ (h^−1^)
Design 1	Chamber 1	16.88	0.99	0.57
	Chamber 2	16.81	0.99	0.53
	Chamber 3	16.07	1.04	0.63
Design 2	Chamber 1	14.83	1.12	1.07
	Chamber 2	14.52	1.15	1.12
	Chamber 3	14.34	1.16	1.10

These discrepancies, depicted in Table 2, clearly illustrate the current problem with quantitative analysis using MSCC when cell loss steadily occurs during long-term cultivation. As evaluating single-cell data manually is highly time-consuming and reliable automated image analysis workflows are not available yet, determining coherent growth rates from colony level is a precondition to conduct systematic studies with high throughput and parallelization in the future. The presented adaptation of our previously developed microfluidic design clearly leads to a consistency of single-cell and colony growth data. Thus, implementing our proposed cell retention concept is a first step towards systematic single-cell growth studies in microfluidic cultivation devices.

## Conclusion

The non-invasive retention of single cells and cultivation of (motile) cells in microfluidic devices always represents a difficult challenge. Especially when coupled with live cell imaging, cellular growth and movement must be spatially restricted, otherwise single-cell cultivation and analysis are not feasible due to constant loss of individual cells. As CHO suspension cells show random movement inside microfluidic cultivation chambers, we tackled the challenge of cell loss by applying a novel cell retention concept based on the introduction of a physical blocking structure into a previously published MSCC device^13^. During the loading procedure cells can be pushed into the cultivation chamber past the barrier, afterwards the barrier prevents cells from escaping. At the same time, single cells can be cultivated for multiple days without any nutrient limitations, although the cross section between supply channel and cultivation chamber which enables diffusive mass exchange was decreased.

By introducing our novel cell retention concept to the field of microfluidic single-cell cultivation, systematic studies of cellular behavior can be performed in a reproducible way without decreased significance due to constant loss of analyzed cells.

Owing to the modular structure of our novel cell retention concept and its simple way of retaining (motile) cells, the principle can be transferred to other cultivation chamber-based designs and thus be applied for the cultivation of other organisms in general. Furthermore, not only single-cell growth studies might be feasible but also a broad variety of taxis studies or analyses of directed migration in restricted compartments are conceivable^26^. Therefore, we believe that our cell retention concept can have a wider field of application than microfluidic cultivation of cells and will pave the way for future applications in the context of basic and biomedical single-cell research.

## Methods

### Microfluidic device fabrication

In a two-layer photolithographic process a silicon wafer, exhibiting the relevant structures as negative relief for subsequent PDMS molding, was fabricated in cleanroom facilities. By applying permonosulphuric acid and demineralised water, the respective 4-inch wafer (MicroChemicals, Germany) was cleaned. In the following, the wafer was spin coated and baked for 15 min at 200 °C for dehydration baking. Afterwards, SU-8 (10) negative photoresist (59% solid, micro resist Technology GmbH, Germany) was applied by spin coating to an 8.5 µm thick layer and pre-backed at 65 °C for 5 min and 95 °C for 10 min. A laser beam written 4-inch photomask (Deltamask, Netherlands) was utilized for the UV exposure step. The exposure time was set to 6 s in vacuum contact mode employing a mask aligner unit (MJB3, Süss MicroTec, Germany). Subsequently, the wafer was post-backed at 65 °C and 95 °C for 10 min and developed by a negative resist developer (mrDev 600, MircoChemicals, Germany). For the second layer, a 8.5 µm thick negative photoresist layer (59% solid) was spin coated onto the wafer. The following steps were alike the first photolithographic procedure. Finally, the wafer was baked at 200 °C for 30 min to seal cracks in the photoresist. To generate a microfluidic cultivation device from the fabricated wafer, PDMS base and curing agent (Sylgard 184 silicone elastomer, Dow Corning Corporation, USA) were mixed in a ratio of 10:1, poured onto the wafer, and degassed for 30 min using an exicator. In a following step, the polymer was cured for 2 h at 80 °C and subsequently cut from the wafer. Applying a biopsy puncher (0.75 mm diameter, Robbins Instruments, USA), inlets and outlets were introduced to the PDMS chip and it was rinsed with isopropanol; likewise, a glass substrate (76 × 26 × 1 mm microscope slides; VWR International GmbH, Germany) was cleaned. Afterwards, PDMS chip and glass substrate were O_2_ plasma activated (Femto Plasma Cleaner, Diener Electronics, Germany) and assembled. To strengthen PDMS-glass bonding, the microfluidic cultivation device was baked for 1 min at 80 °C.

### Fluorescein trace substance experiments

To quantify diffusive mass exchange duration between supply channel and cultivation chamber for both chamber designs, ethanol-dissolved fluorescein (Macrolex yellow, Lanxess, Germany) was applied as trackable trace substance. The microfluidic cultivation device was primed with ethanol and subsequently fluorescein was flushed into the supply channels with a flow rate of 2 µL min^−1^. For quantification, fluorescence images were exported as TIFF images and grey values were analyzed: The average grey value of the supply channel and cultivation chamber respectively after 900 s were set as 100% and the grey value at 0 s was set as 0%, so that relative fluorescence increase could be determined for every image. The analysis was performed for one cultivation chamber.

### CFD simulations

Computational fluid dynamics simulations (CFD) were performed to analyze nutrient supply inside the cultivation chamber designs as described before^13,27^. For the CFD simulations COMSOL Multiphysics Version 5.6 (COMSOL AB, Sweden) was applied. Glucose concentration profiles during MSCC, assuming a steady state with 181 cells inside the chamber with a constant glucose uptake rate of 3800 nmol per 10^6^ cells and day, were simulated under consideration of the respective cultivation chamber geometry. Cellular uptake was assumed to be homogeneously distributed over the whole cultivation chamber. Glucose concentration inside the supply channels was set to 45 mol m^−3^.

### Cell culture and medium

CHO-K1 cells (strain ATCC 61-CCL, adapted to growth in suspension) were cultivated in commercially available medium (TXC6D, Xell AG, Germany), supplemented with 6 mM glutamine, in 125 mL shake flasks (Flat Base, TriForest, USA) with a working volume of 60 mL at 37 °C, 5% CO_2_, 80% humidity, and 185 rpm on an orbital shaker (ES-X, Kühner AG, Switzerland). The pre-culture was passaged in the exponential phase two or three times before any cultivation experiment to guarantee reproducibility. For MSCC, the applied cell culture medium was mixed with already conditioned medium obtained from the exponential growth phase of a shake flask cultivation in a ratio of 1:1 to mimic metabolite and substrate conditions of a standard batch cultivation.

### Cell seeding and cultivation

For cell seeding, the microfluidic cultivation device was mounted onto the stage of the live cell imaging microscope. Seeding itself was performed manually using a cell suspension containing single-use syringe with a cell density of 3 to 5 × 10^6^ cells mL^−1^. For Design 1, the cell suspension was moved back and forth through the supply channels to seed cells into the adjacent cultivation chambers. When enough cells randomly entered the cultivation chambers, remaining cells were flushed out of the supply channels using cultivation medium. For Design 2, air was manually introduced into the supply channels in a controlled way (Supplementary Fig. S2, Supplementary Video S1), so that one channel was blocked and subsequent cell suspension flow was directed through the adjacent cultivation chamber. This way, cells were pushed through the narrow entrance of the respective chambers. This procedure must be performed gently to not shear the cells while forcing them past the barrier structure. Again, remaining cells were flushed out of the supply channels by cultivation medium. Following on cell loading, the microfluidic cultivation device was constantly perfused with medium with a flow rate of 2 µL min^−1^ applying low pressure syringe pumps (neMESYS, CETONI, Germany) and 20 mL single-use syringes. Cultivation temperature and CO_2_ atmosphere were kept constant at 37 °C respectively 5% by a microscope incubator system and an additional CO_2_ incubation chamber (OKO Touch, Okolab S.R.L.; H201-K-FRAME GS35-M, Okolab S.R.L.).

### Live cell imaging

In order to resolve cellular behavior with a high spatial and temporal resolution, an automated live cell imaging microscope (Nikon Eclipse Ti2, Nikon Instruments, Germany) was applied. Using a 40 × objective, every 20 min phase contrast microscopy images of all relevant positions on the microfluidic cultivation device were recorded (NIS Elements AR 5.20.01 Software, Nikon Instruments, Germany).

### Growth and t_D_ analysis

The specific growth rate µ_max_ and doubling time t_D_ were analyzed to compare growth characteristics of the captured microcolonies. Cell number inside a cultivation chamber was enumerated manually every 5 h by analyzing the microscope images. The growth rate was identified graphically by determining the slope of the linear regression from the semi-logarithmical plot using OriginPro (OriginPro 2020b 9.7.5.184, OriginLab Corporation, USA). To assess single-cell doubling times, cells were tracked manually over multiple generations and the duration between two cell divisions was determined.

## Supplementary Information

Supplementary Figures.

Supplementary Video S1.

Supplementary Video S2.

Supplementary Video S3.

Supplementary Video S4.

Supplementary Video S5.

## Data Availability

The data presented in this study are available on request from the corresponding author. The data are not publicly available due to the size of the respective image data (multiple gigabytes).

## References

[1] Grünberger A (2012). A disposable picolitre bioreactor for cultivation and investigation of industrially relevant bacteria on the single cell level. Lab Chip.

[2] Grünberger A, Wiechert W, Kohlheyer D (2014). Single-cell microfluidics: Opportunity for bioprocess development. Curr. Opin. Biotechnol..

[3] Grünberger A (2015). Spatiotemporal microbial single-cell analysis using a high-throughput microfluidics cultivation platform. Cytom. A.

[4] Graham PJ, Riordon J, Sinton D (2015). Microalgae on display: A microfluidic pixel-based irradiance assay for photosynthetic growth. Lab Chip.

[5] Groisman A (2005). A microfluidic chemostat for experiments with bacterial and yeast cells. Nat. Methods.

[6] Rowat AC, Bird JC, Agresti JJ, Rando OJ, Weitz DA (2009). Tracking lineages of single cells in lines using a microfluidic device. Proc. Natl. Acad. Sci. U.S.A..

[7] Luke CS (2016). A microfluidic platform for long-term monitoring of algae in a dynamic environment. ACS Synth. Biol..

[8] Ullman G (2013). High-throughput gene expression analysis at the level of single proteins using a microfluidic turbidostat and automated cell tracking. Philos. Trans. R. Soc. Lond. Ser. B Biol. Sci..

[9] Demming S (2011). Disposable parallel poly(dimethylsiloxane) microbioreactor with integrated readout grid for germination screening of *Aspergillus **ochraceus*. Biomicrofluidics.

[10] Kim HS, Devarenne TP, Han A (2015). A high-throughput microfluidic single-cell screening platform capable of selective cell extraction. Lab Chip.

[11] Ziółkowska K (2013). Long-term three-dimensional cell culture and anticancer drug activity evaluation in a microfluidic chip. Biosens. Bioelectron..

[12] Tokárová V (2021). Patterns of bacterial motility in microfluidics-confining environments. Proc. Natl. Acad. Sci..

[13] Schmitz J (2021). Development and application of a cultivation platform for mammalian suspension cell lines with single-cell resolution. Biotechnol. Bioeng..

[14] Marques MP, Szita N (2017). Bioprocess microfluidics: Applying microfluidic devices for bioprocessing. Curr. Opin. Chem. Eng..

[15] Walsh G (2018). Biopharmaceutical benchmarks 2018. Nat. Biotechnol..

[16] Zhu MM, Mollet M, Hubert RS, Kyung YS, Zhang GG (2017). Industrial Production of Therapeutic Proteins: Cell Lines, Cell Culture, and Purification.

[17] Schmitz J, Hertel O, Yermakov B, Noll T, Grünberger A (2021). Growth and eGFP production of CHO-K1 suspension cells cultivated from single cell to laboratory scale. Front. Bioeng. Biotechnol..

[18] Lecault V (2011). High-throughput analysis of single hematopoietic stem cell proliferation in microfluidic cell culture arrays. Nat. Methods.

[19] Dadgar N (2020). A microfluidic platform for cultivating ovarian cancer spheroids and testing their responses to chemotherapies. Microsyst. Nanoeng..

[20] Eu Y-J, Park H-S, Kim D-P, Wook Hong J (2014). A microfluidic perfusion platform for cultivation and screening study of motile microalgal cells. Biomicrofluidics.

[21] Dettinger P (2018). Automated microfluidic system for dynamic stimulation and tracking of single cells. Anal. Chem..

[22] Gómez-Sjöberg R, Leyrat AA, Pirone DM, Chen CS, Quake SR (2007). Versatile, fully automated, microfluidic cell culture system. Anal. Chem..

[23] Delincé MJ (2016). A microfluidic cell-trapping device for single-cell tracking of host-microbe interactions. Lab Chip.

[24] Halperin SO (2014). A massively parallel microfluidic device for long-term visualization of isolated motile cells. Microfluid. Nanofluid..

[25] Kolnik M, Tsimring LS, Hasty J (2012). Vacuum-assisted cell loading enables shear-free mammalian microfluidic culture. Lab Chip.

[26] Höving AL (2021). Human blood serum induces p38-MAPK- and Hsp27-dependent migration dynamics of adult human cardiac stem cells: Single-cell analysis via a microfluidic-based cultivation platform. Biology.

[27] Westerwalbesloh C, Grünberger A, Wiechert W, Kohlheyer D, von Lieres E (2017). Coarse-graining bacteria colonies for modelling critical solute distributions in picolitre bioreactors for bacterial studies on single-cell level. Microb. Biotechnol..

