# Supplementary material for: Reliable cell retention of mammalian suspension cells in microfluidic cultivation chambers
Source: Sci Rep. 2023 Mar 8;13:3857. doi: 10.1038/s41598-023-30297-5 (PMC9995442; doi:10.1038/s41598-023-30297-5)
Supplement: Supplementary file 1 — Supplementary Figures. [file 41598_2023_30297_MOESM1_ESM.docx]

Supplementary Information

Reliable Cell Retention of Mammalian Suspension Cells in Microfluidic Cultivation Chambers

Julian Schmitz, Birgit Stute, Sarah Täuber, Dietrich Kohlheyer, Eric von Lieres, Alexander Grünberger*


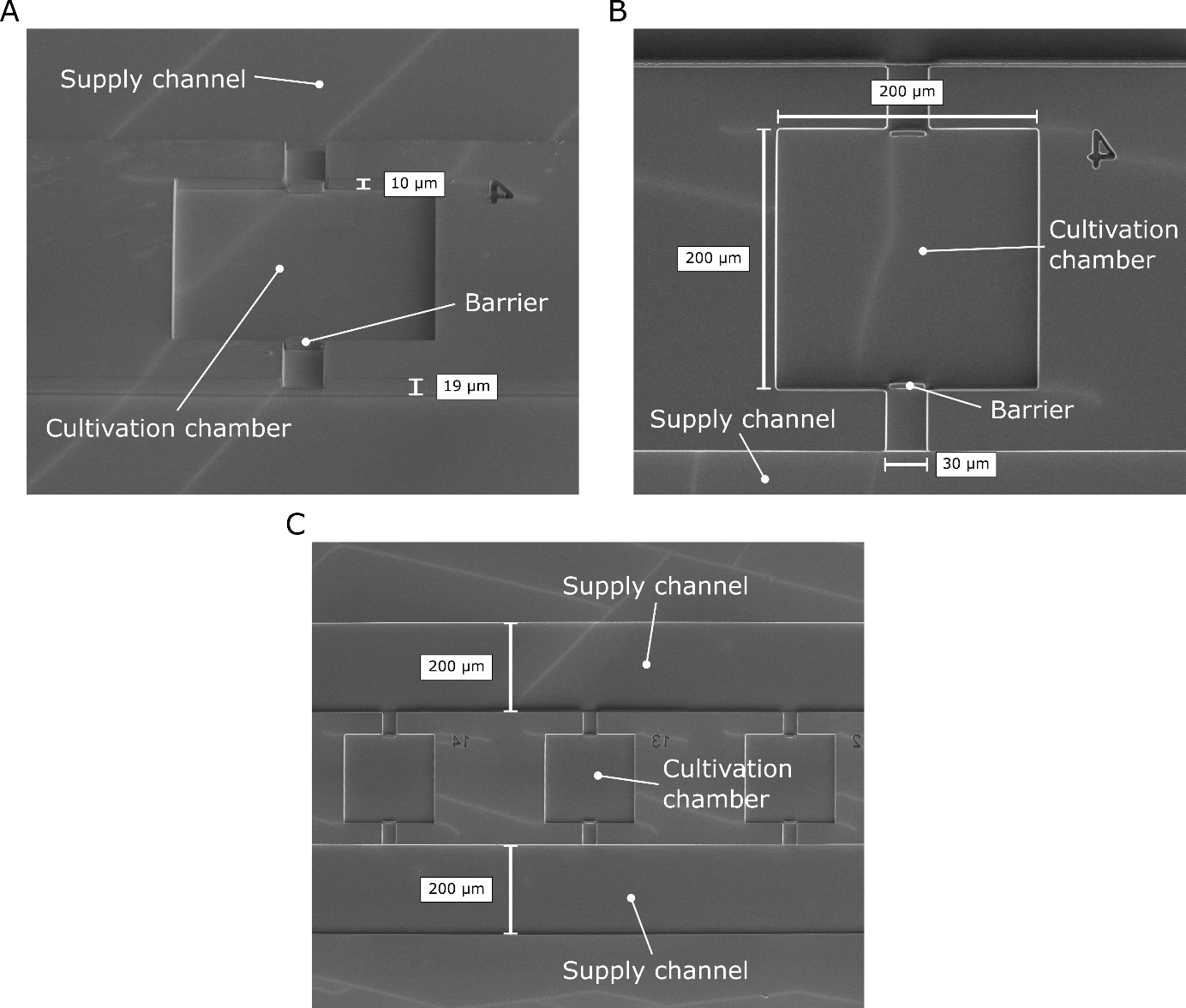


**Figure S1.** Scanning electron microscope (SEM) images of the microfluidic cultivation device with PDMS barrier in the cultivation chamber’s entrance. (A) The approx. height of the cultivation chamber is 10 µm while the supply channel is approx. 19 µm high. (B) The base area of the cultivation chamber is 200 x 200 µm^2^ and the chamber entrance has a width of 30 µm. (C) The displayed image section shows three cultivation chambers that are lined up along the adjacent supply channels. The channels have a width of 200 µm.


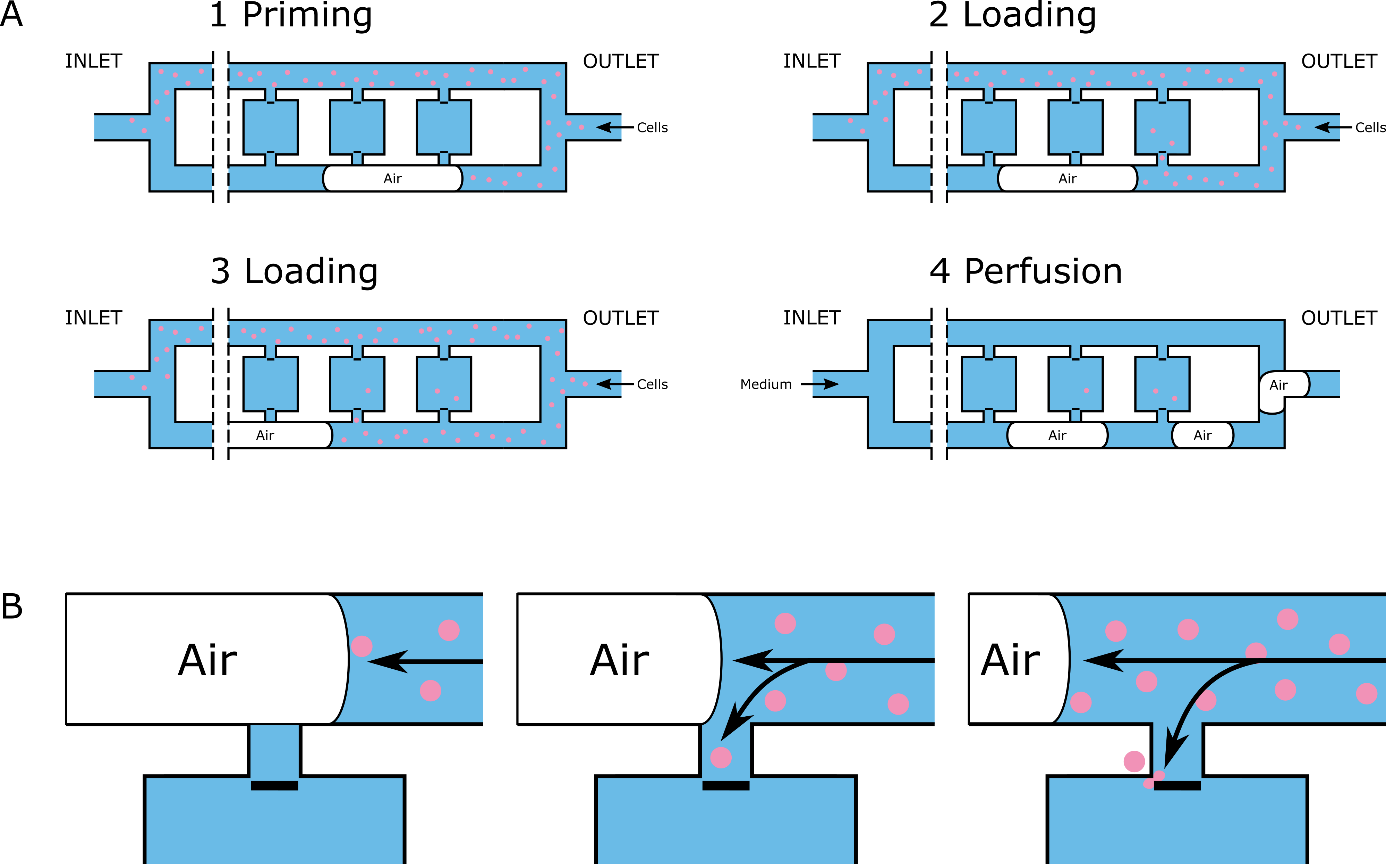


**Figure S2.** Loading procedure to capture cells inside the MSCC device with the novel trapping concept. (A) The MSCC device is manually flushed with cell suspension from the outlet side using a single-use syringe. By introducing air into the supply channels, one adjacent channel is blocked so that the cultivation chambers can directly be flushed with cell suspension. After sufficient loading, medium is pumped through the MSCC device from the inlet side so that remaining air is pushed out of the channels and constant perfusion can be established. (B) Zoom-in of the chamber’s entrance. Once the entrance is no longer blocked by air, the flow is directed into the cultivation chamber. Due to the increased pressure of manual loading, cells are pushed through the narrow gap between barrier and the walls of the respective cultivation chamber.


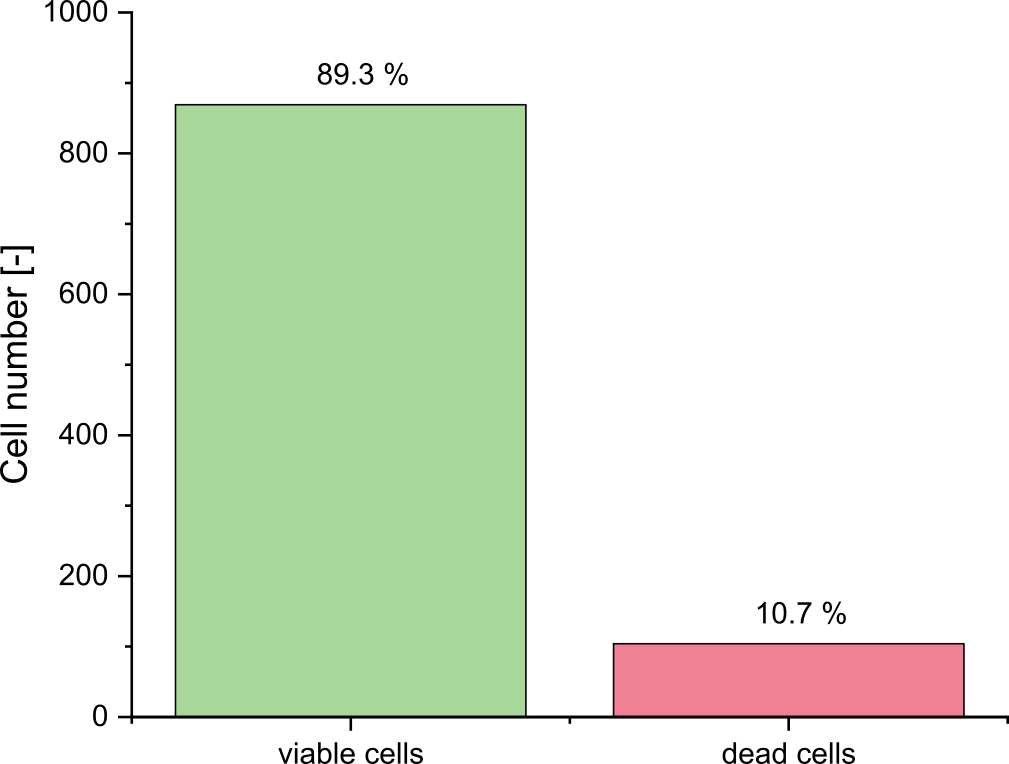


**Figure S3.** Analysis of cellular viability after cell loading procedure. In this experiment, a cell suspension with a viable cell density of 7.7 x 10^6^ cells mL^-1^ was used for cell loading to enforce a high repetition of potentially damaging events while the cells traverse the chamber’s barrier. After loading the device, cells were incubated for 4 h before live/death staining was performed by flushing the array with a trypan blue solution. This way, cells with a damaged membrane (dead cells) were stained and cells with an intact membrane (viable cells) remained unstained. In addition, already heavily disintegrated cells were identified as dead cells manually. Out of the 1077 loaded cells 89.3 % were viable subsequent to cell loading while only 10.7 % appeared to be dead.


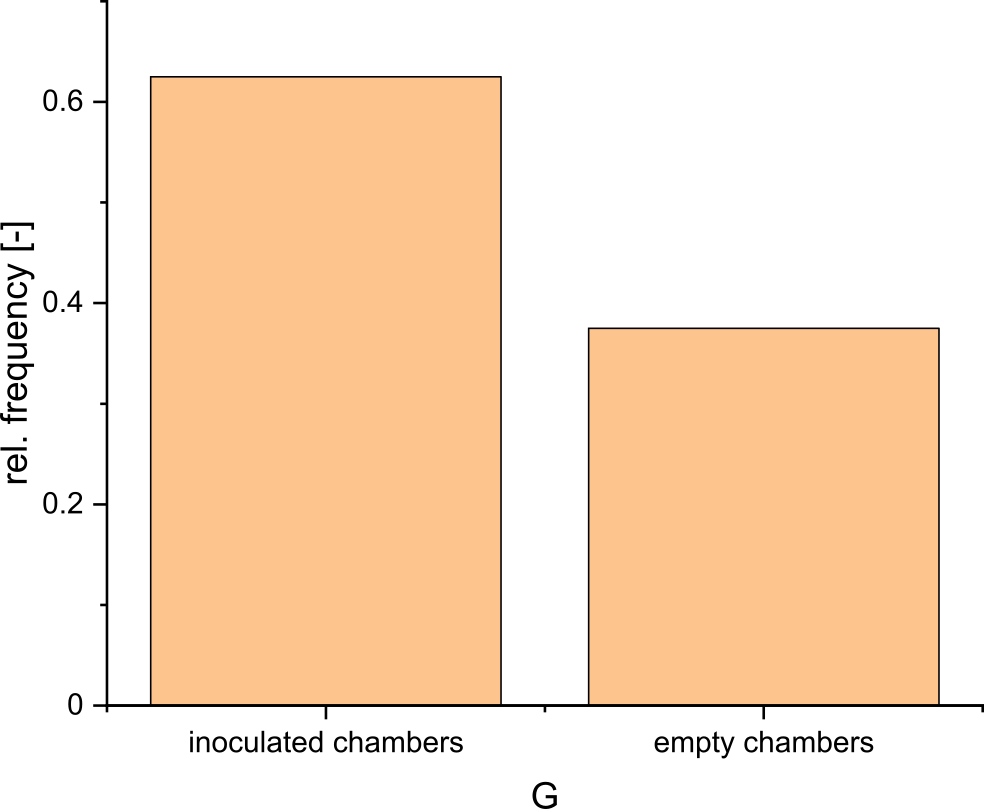


**Figure S4.** Proportion of inoculated and empty chambers after a representative loading procedure. After loading four cultivation arrays on a microfluidic cultivation device with a loading cell density of 3.59 x 10^6^ cells mL^‑1^ the relative frequency of occurrence for inoculated chambers and empty chambers was determined manually. Out of the 240 potentially loadable chambers 150 chambers (62.5 %) showed one ore more cells after performing the loading procedure while 37.5 % remained unloaded.


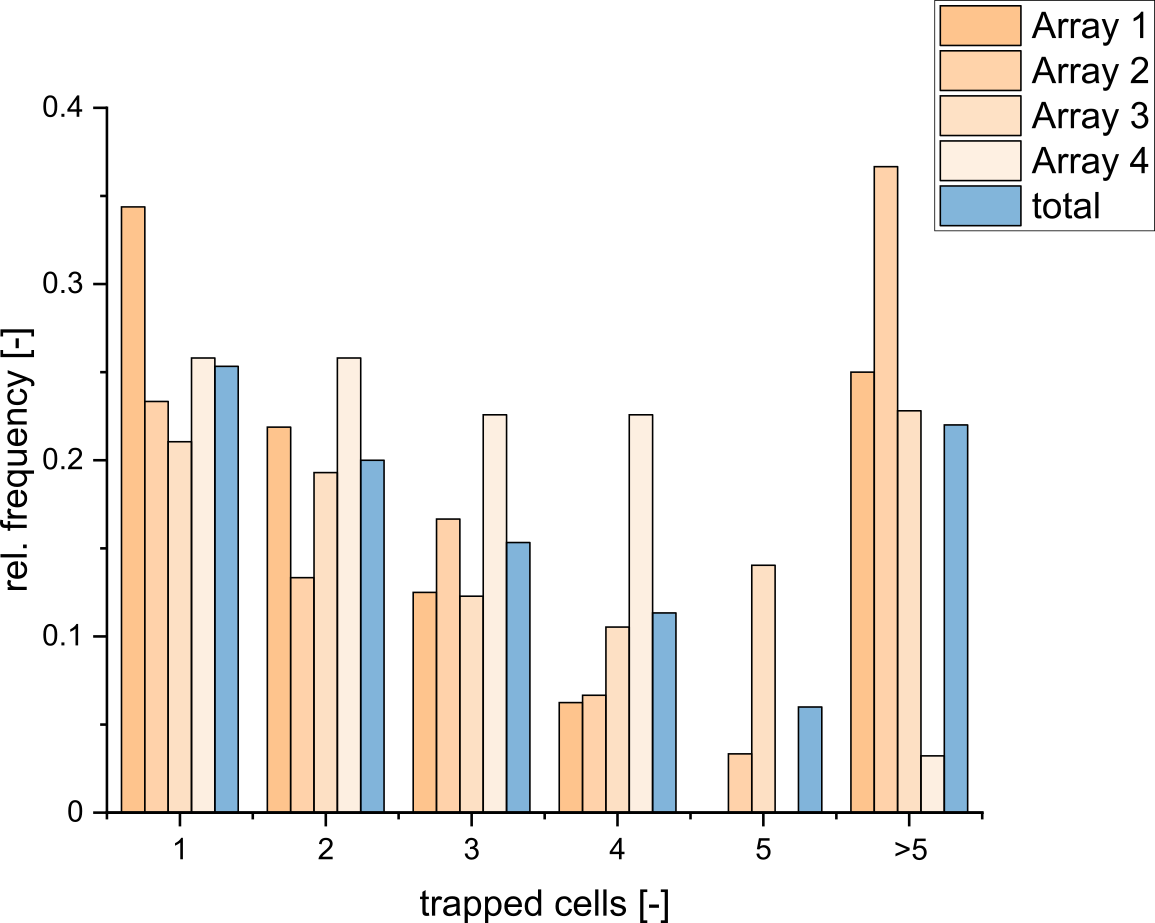


**Figure S5.** Loading efficiency divided by cultivation arrays after a representative loading procedure. After loading four cultivation arrays on one microfluidic cultivation device with a loading cell density of 3.59 x 10^6^ cells mL^‑1^ the relative frequency of occurrence for chambers inoculated with 1, 2, 3, 4, 5, and more than 5 cells was determined manually for every cultivation array individually as well as cumulatively for all arrays (total). Concerning the chambers with less than 6 cells, the frequency of occurrence of chambers with one cell is the highest and it decreases gradually with the number of initial cells per chamber for all arrays. The cumulative loading efficiency reflects this characteristic as well.


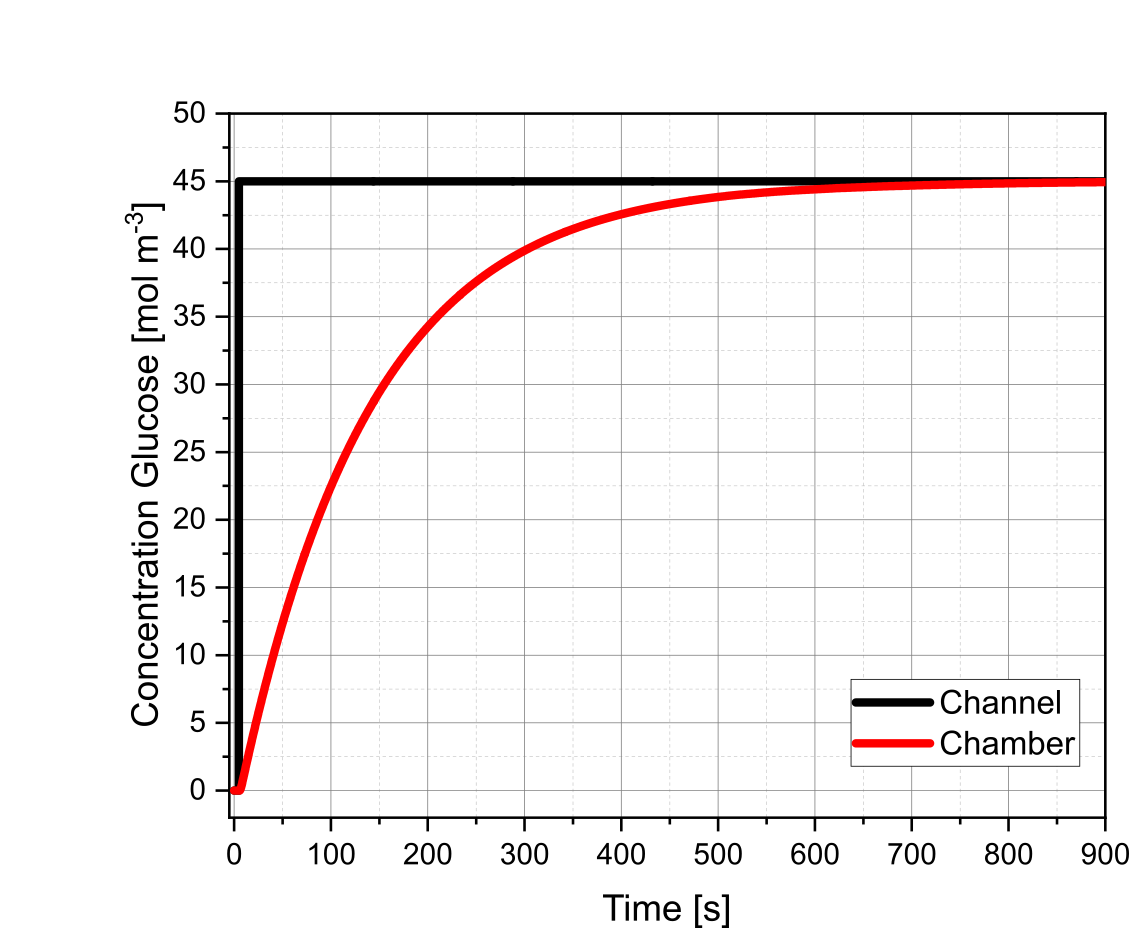


**Figure S6.** CFD simulation of glucose concentration inside the supply channels and the cultivation chamber after a quick medium change from 0 mol m^-3^ to 45 mol m^-3^ glucose for Design 2. The CFD simulation was performed to support the experimental characterization of the microfluidic device concerning the medium exchange duration and was conducted as described before [13, 27]. Like already shown by the experimental data the diffusive mass exchange is also estimated to take approx. 600 s until equilibrium between the supply channel’s concentration and the cultivation chamber’s concentration is reached based on the CFD simulations and therefore resembles the experimental data.
